# Neuro-Sliding Control for Underwater ROV’s Subject to Unknown Disturbances

**DOI:** 10.3390/s19132943

**Published:** 2019-07-04

**Authors:** Luis Govinda García-Valdovinos, Fernando Fonseca-Navarro, Joanes Aizpuru-Zinkunegi, Tomas Salgado-Jiménez, Alfonso Gómez-Espinosa, José Antonio Cruz-Ledesma

**Affiliations:** 1 Centro de Ingeniería y Desarrollo Industrial, Dirección de Energía, Av. Playa Pie de la Cuesta 702, Querétaro 76125, Mexico; 2 Tecnologico de Monterrey, Escuela de Ingeniería y Ciencias, Ave. Epigmenio González 500, Fracc. San Pablo, Querétaro 76130, Mexico; 3 Escuela Politécnica Superior, Universidad Mondragón, 20500 Gipuzkoa, País Vasco, Spain

**Keywords:** backpropagation neural network, high order sliding mode control, underwater ROVs

## Abstract

Proposed in this paper is a model-free and chattering-free second order sliding mode control (2nd-SMC) in combination with a backpropagation neural network (BP-NN) control scheme for underwater vehicles to deal with external disturbances (i.e., ocean currents) and parameter variations caused, for instance, by the continuous interchange of tools. The compound controller, here called the neuro-sliding control (NSC), takes advantage of the 2nd-SMC robustness and fast response to drive the position tracking error to zero. Simultaneously, the BP-NN contributes with its capability to estimate and to compensate online the hydrodynamic variations of the vehicle. When a change in the vehicle’s hydrodynamics occurs, the 2nd-SMC may no longer be able to compensate for the variations since its feedback gains are tuned for a different condition; thus, in order to preserve the desired performance, it is necessary to re-tune the feedback gains, which a cumbersome and time consuming task. To solve this, a viable choice is to implement a BP-NN control scheme along with the 2nd-SMC that adds or removes energy from the system according to the current condition it is in, in order to keep, or even improve, its performance. The effectiveness of the proposed compound controller was supported by experiments carried out on a mini-ROV.

## 1. Introduction

Underwater robots are nonlinear systems that operate in complex marine environments. They are subject to ocean currents that affect their performance by following a required trajectory or keeping a desired position while carrying out specific tasks. Moreover, their performance is also affected by the addition or removal of tools like robot arms and/or measurement instruments, among others, during the execution of a task because their weight and hydrodynamic parameters change accordingly. These changes are considered as external perturbations that need to be compensated for in order to achieve a desired performance [1,2].

Much research has been conducted in the area of underwater robots to tackle the problem described above. In reference [1], Zhang and Zhu proposed a model-based adaptive first order sliding mode control based on local recurrent neural networks. The approach transforms the trajectory tracking error system into an affine nonlinear system and uses a local recurrent neural network to approximate unknown items online for the adaptive trajectory tracking control. To reduce the chattering amplitude, the sliding mode gain is adjusted with an exponential function. Sun et al. [2] proposed a model-based conventional sliding mode control where the discontinuous function was substituted for an adaptation law to eliminate the chattering. Gao et al. [3] presented a single hidden layer neural network together with a conventional PI control to compensate for the uncertainties in both the dynamic model and the Jacobian matrix. Furthermore, Cui et al. [4] proposed a first order sliding mode control wherein the signum function was substituted for a boundary layer to considerably reduce the chattering at the cost of stability and robustness. Yang et al. [5] proposed a second order sliding mode control approach to solve the problem of chattering together with a disturbance observer based on recurrent hermite neural networks to estimate the model uncertainties and composite disturbances. In addition, finite-time convergence was achieved; however, all of the progress was only presented in the simulation results. Guo et al. [6] proposed a first-order sliding-mode control based on a linear-in parameter neural network to deal with the unknown dynamics and the external environmental disturbances as well as the introduction of an exact differentiator to estimate the velocities of an AUV. Similarly, only the simulation results were presented. In reference [7], Gao et al. presented the simulation results of a visual servoing controller combined with a single-hidden-layer neural network, in conjunction with a first order sliding mode controller, to compensate for dynamic uncertainties and external disturbances. Liu et al. [8] presented a robust adaptive self-organizing neuro-fuzzy tracking control for underwater vehicles with system uncertainties and an unknown dead-zone non linearity. The robust adaptive part was composed of a sliding mode control with a self-organizing neuro-fuzzy network. The simulation results showed the good performance of the presented controller. Gao et al. [9] proposed a visual servo control together with a model-free conventional sliding mode control and a neural network to compensate for the dynamic uncertainties. The discontinuous signum function was replaced by a saturation function to eliminate the chattering phenomenon. One advantage is that the neural network weights can be calculated online, which is very useful for real time applications and only the simulation results were given. In reference [10], Elmokadem et al. presented the simulation results of a first order terminal sliding mode control for the tracking trajectory of underwater vehicles. Three kinds of terminal sliding mode were presented, all of which guaranteed finite-time convergence of the position tracking errors; however, to eliminate the chattering, the signum function was replaced by a saturation function, at the cost of decreasing robustness and performance. Londhe et al. [11] presented the simulation results of a model-based first order sliding mode control for a linearized model to control the lateral motion of an AUV. The sliding mode controller was tailored with an algorithm able to estimate online and compensate for the unpredictable disturbances. In reference [12], García-Valdovinos et al. proposed a smooth but robust model-free second order sliding mode controller for underwater vehicles subject to ocean currents. Experimental results were given for a system with one degree of freedom. In reference [13], Hernandez-Alvarado et al. proposed a neural network-based self-tuning PID control for underwater vehicles. In this work, the PID feedback gains were auto-tuned by means of a recurrent backpropagation neural network according to the changing conditions; and the controller was validated through a series of experiments carried out on a mini-ROV.

The second order sliding mode control (2nd-SMC) technique has been shown to be a suitable approach to deal with and compensate for disturbances and parametric uncertainties provided the feedback gains are carefully tuned for a specific operating condition [14]. However, if the ocean currents and/or the vehicle’s parameters change during the execution of the task, the initial set of feedback gains may no longer preserve the desired performance since they were tuned under different operating conditions. To attain the desired performance, the 2nd-SMC feedback gains have to be re-tuned to the new operating conditions, which is a cumbersome and time-consuming activity. To address this problem, a backpropagation neural network (BP-NN) was proposed in this paper to compensate for the disturbances by online calculation of the necessary control signal amount to preserve the desired performance. On one hand, the 2nd-SMC acts as a “gross” control that rapidly drives the position tracking error very close to zero. On the other hand, the BP-NN control acts as a “fine” control that does the same, but gradually. The feasibility and effectiveness of the proposed approach were demonstrated with experiments carried out on a mini-ROV.

The rest of this paper is organized as follows. Section 2 presents and describes the standard mathematical models for underwater robots. Section 3 is devoted to describing in detail the structure and properties of the proposed neuro-sliding control, called the NSC scheme. Section 4 explains the electromechanical architecture of the mini-ROV used for the experiments in detail. Two sets of exhaustive experiments were developed; and the results were analyzed and discussed taking into account two criterions to evaluate the performance. Finally, Section 5 presents our concluding remarks on the NSC scheme.

## 2. Mathematical Model

**Figure 1:**
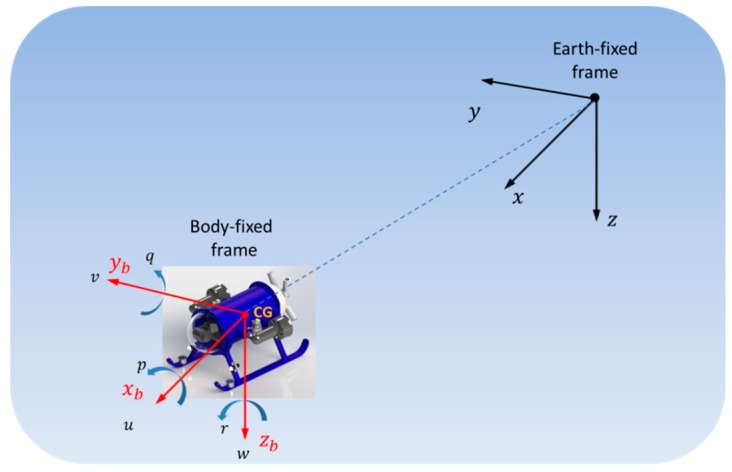
Coordinated frames used in the modeling and analysis of underwater vehicles.

The kinematic and dynamic models of an underwater vehicle [15] can be expressed in terms of an Earth-fixed frame xyz and a body-frame $x_{b}y_{b}z_{b}$ attached to the vehicle’s center of gravity (CG), as shown in Figure 1.

The kinematic equation of motion is given by:

(1) $$\dot{\eta }=J\left(\eta \right)\nu$$

where the pose vector $\eta ={\left[\begin{array}{ccc} x & y & z\; \end{array}\begin{array}{ccc} \phi & \theta & \psi \end{array}\right]}^{T}\in {\mathbb{R}}^{6}$ contains the global position $\eta_{1}={\left[\begin{array}{ccc} x & y & z \end{array}\right]}^{T}$ and the Euler angles $\eta_{2}={\left[\begin{array}{ccc} \phi & \theta & \psi \end{array}\right]}^{T}$ expressed in the Earth-fixed frame; the velocity vector $\nu ={\left[\begin{array}{ccc} u & v & w\; \end{array}\begin{array}{ccc} p & q & r \end{array}\right]}^{T}\in {\mathbb{R}}^{6}$ is composed of the linear velocity vector $\nu_{1}={\left[\begin{array}{ccc} u & v & w \end{array}\right]}^{T}$ and the angular velocity vector $\nu_{2}={\left[\begin{array}{ccc} p & q & r \end{array}\right]}^{T}$, both according to the body-fixed frame; and the matrix J(η) is the matrix that relates the body-fixed vehicle’s velocities to the Earth-fixed frame pose rates. 

The hydrodynamic model is given by

(2) $$M\dot{\nu }+C\left(\nu \right)\nu +D\left(\nu \right)\nu +g\left(\eta \right)=\tau$$

formed by the vectors $\nu \in {\mathbb{R}}^{n}$ and $\eta \in {\mathbb{R}}^{n}$ previously defined above. Matrices $M\in {\mathbb{R}}^{n\times n}$, $C\in {\mathbb{R}}^{n\times n}$, and $D\in {\mathbb{R}}^{n\times n}$ denote the matrix of inertia (including added mass), the matrix of Coriolis and centripetal terms (including added mass), and the damping matrix, respectively. Vectors $g\in {\mathbb{R}}^{n}$ and $\tau \in {\mathbb{R}}^{n}$ represent the restoring force and moment vector produced by gravity and buoyancy and the vector with the control inputs, respectively.

To eliminate ν and $\dot{\nu }$ from Equation (2), the hydrodynamic model Equation (2) can be rewritten in the Earth-fixed frame by applying the next kinematic transformations (assuming that J(η) is non-singular) [15,16]:

(3) $$\dot{\eta }=J\left(\eta \right)\nu \;\;\;\;\;\;\;\;\;\;\;\;\;\;\Leftrightarrow \;\;\;\;\;\;\;\;\;\;\nu =J^{-1}\left(\eta \right)\dot{\eta }$$

(4) $$\ddot{\eta }=J\left(\eta \right)\dot{\nu }\;+\dot{J}\left(\eta \right)\nu \;\;\;\;\Leftrightarrow \;\;\;\;\;\dot{\nu }=J^{-1}\left(\eta \right)\;\left[\ddot{\eta }-\dot{J}\left(\eta \right)\nu \right]$$

After some calculations, the Earth-fixed expression is given below [15]

(5) $$M_{\eta }\left(\eta \right)\ddot{\eta }+C_{\eta }\left(\nu ,\eta \right)\dot{\eta }+D_{\eta }\left(\nu ,\eta \right)\dot{\eta }+g_{\eta }\left(\eta \right)=\tau_{\eta }$$

with

(6) $$M_{\eta }\left(\eta \right)=J^{-T}\left(\eta \right)MJ^{-1}\left(\eta \right)\;C_{\eta }\left(\nu ,\eta \right)=J^{-T}\left(\eta \right)\left[C\left(\nu \right)-MJ^{-1}\left(\eta \right)\;\dot{J}\left(\eta \right)\right]\;J^{-1}\left(\eta \right)\;D_{\eta }\left(\nu ,\eta \right)=\;J^{-T}\left(\eta \right)\;D\left(\nu \right)J^{-1}\left(\eta \right)\;\;g_{\eta }\left(\eta \right)=J^{-T}\left(\eta \right)g\left(\eta \right)\;\;\tau_{\eta }\left(\eta \right)=J^{-T}\left(\eta \right)\tau$$

## 3. Control Design

**Figure 2:**
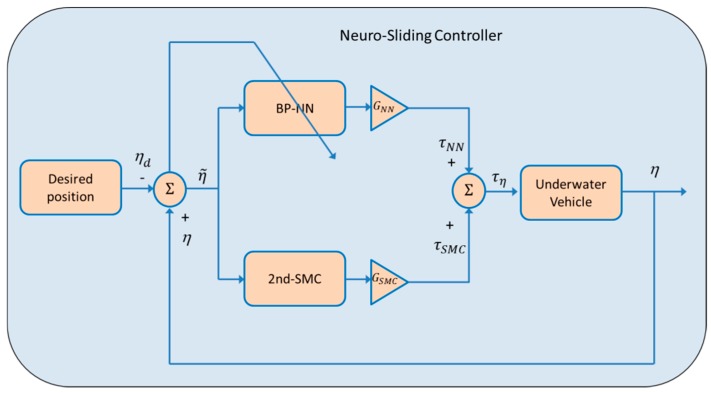
Block diagram of the neuro-sliding controller (NSC).

As briefly mentioned earlier, the proposed control scheme is composed of two control laws: a second order sliding mode control (2nd-SMC) and a self-tuning backpropagation neural network control (BP-NN). The compound control uses the advantages of both controllers, the fastness of the 2nd-SMC to drive the vehicle to the desired position, and the BP-NN’s ability to counteract disturbances online to minimize the position tracking error within a tolerable time interval of about 10 to 20 s. A block diagram of the compound control scheme is shown in Figure 2.

According to Equation (5) and Figure 2, $\tau_{\eta }=\tau_{SMC}+\tau_{NN}$. This means that the compound control signal $\tau_{\eta }$ is the sum of the control signal calculated separately. The control design for each control law is given below. 

### 3.1. Second Order Sliding Mode Control (2nd-SMC)

According to Equation (5) and Figure 2, it is possible to parameterize linearly as the product of the regressor $Y\left(\eta ,\dot{\eta },\ddot{\eta }\right)\in {\mathbb{R}}^{n\times p}$ and the vector $\theta \in {\mathbb{R}}^{p}$. Consequently, the parametrization $Y\left(\eta ,\dot{\eta },\ddot{\eta }\right)\theta$ can be written in terms of a nominal reference ${\dot{\eta }}_{r}$, and its time derivative ${\ddot{\eta }}_{r}$ as follows:

(7) $$M_{\eta }\left(\eta \right){\ddot{\eta }}_{r}+C_{\eta }\left(\nu ,\eta \right){\dot{\eta }}_{r}+D_{\eta }\left(\nu ,\eta \right){\dot{\eta }}_{r}+g_{\eta }\left(\eta \right)=Y\left(\eta ,\dot{\eta },{\dot{\eta }}_{r},{\ddot{\eta }}_{r}\right)\theta$$

Subtracting Equation (7) from both sides of Equation (5) yields the open-loop error dynamics equation:

(8) $$M_{\eta }\left(\eta \right){\dot{s}}_{r}+C_{\eta }\left(\nu ,\eta \right)s_{r}+D_{\eta }\left(\nu ,\eta \right)s_{r}=\tau_{SMC}-Y_{r}\left(\eta ,\dot{\eta },{\dot{\eta }}_{r},{\ddot{\eta }}_{r}\right)\theta$$

where $\tau_{\eta }$ is substituted for $\tau_{SMC}$ and $s_{r}=\dot{\eta }-{\dot{\eta }}_{r}$ represents the extended error. The problem of control design for the open-loop error dynamics system (Equation (8)) is to find $\tau_{SMC}$ so that it converges exponentially when $Y_{r}\left(\eta ,\dot{\eta },{\dot{\eta }}_{r},{\ddot{\eta }}_{r}\right)\theta$ is not available.

#### 3.1.1. Nominal Reference

Now, consider the following nominal reference

(9) $${\dot{\eta }}_{r}={\dot{\eta }}_{d}-\alpha \tilde{\eta }+s_{d}-K_{i}\overset{t}{\underset{0}{\int }}sign\left(s_{\eta }\right)d\sigma$$

where $\tilde{\eta }=\eta -\eta_{d}$ denotes the position tracking error and $\eta_{d}$ denotes the desired trajectory; α,$K_{i}\in {\mathbb{R}}^{n\times n}$ are gain matrices; sign(x) stands for the entry wise signum function of vector x; and

(10) $$s=\;\dot{\tilde{\eta }}+\alpha \tilde{\eta },\;\;\;s_{d}=s\left(t_{0}\right)e^{-kt},\;\;\;\;s_{\eta }=s-s_{d}$$

with κ>0. Considering Equation (9), the extended error $s_{r}$ can be rewritten as follows

(11) $$s_{r}=s_{\eta }+K_{i}\overset{t}{\underset{0}{\int }}sign\left(s_{\eta }\right)d\sigma$$

#### 3.1.2. Sliding Mode Control

Take into account the next control law [12]:

(12) $$\tau_{SMC}=-K_{d}s_{r}$$

where $K_{d}\in {\mathbb{R}}^{n\times n}$ is a gain matrix. The control does neither require any knowledge of the dynamics nor any parameters of the system. Major information about the controller can be found in [12].

### 3.2. Self-Tuning Backpropagation Neural Network Control 

In this work, the self-tuning backpropagation neural network control, shown in Figure 2 was implemented. The neural network controller was used to regulate the process, the net parameters were adjusted to improve the system performance, and no previous training was required. An additional reference for the controller can be found in [16].

**Figure 3:**
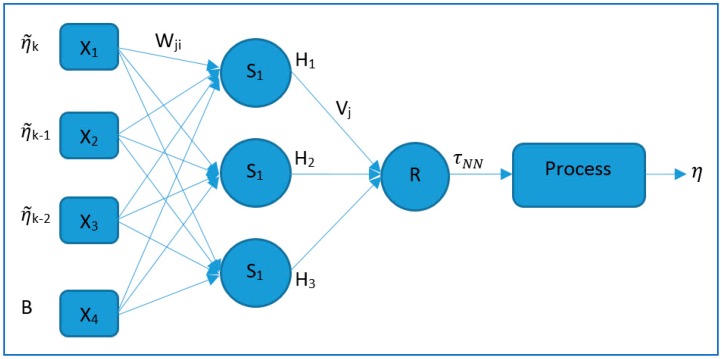
Recurrent neural network with three perceptron-layers.

A recurrent neural network composed of three perceptron-layers was selected. As shown in Figure 3, the present error as well as two past position tracking errors (${\tilde{\eta }}_{k}$, $\tilde{\eta }$*_k_*_−1_, $\tilde{\eta }$*_k_*_−2_), respectively, were used by the input layer (X_1_, X_2_, X_3_). Additionally, a bias unit, B, was incorporated into the input X_4_, which stores the value of 1. The use of a bias unit in the neural network increases the capability of the network to adjust to the system.

In this case, the net output error was used to present the position tracking error,$\;{\tilde{\eta }}_{k}$, as the difference between the process output, $\eta_{k}$, and the reference value, $\eta_{dk}$, instead of as a comparison between the desired and present net output:

(13) $${\tilde{\eta }}_{k}=\eta_{k}-\eta_{dk}$$

Each of the hidden layer neurons computes the weighted sum *S_j_* based on the inputs *X_i_* with its weight coefficient *W_ji_*:

*S_j_* = *Σ* (*X_i_* · *W_ji_*) (14)

In the hidden layer, for each neuron, the output *H_j_* is calculated by the sigmoid function applied to the weighted sum *S_j_*:

(15) $$H_{j}=\frac{1}{1+e^{-S_{j}}}$$

Similarly, for the output layer neuron, the weighted sum *R* over the outputs *H_j_* and its weight coefficients *V_j_* were calculated:

*R* = Σ (*H_j_ · V_j_*) (16)

For the output layer neuron, the net output $\tau_{NN}$ was calculated by the sigmoid function applied to the weighted sum *R*, and scaled by a positive gain factor $G_{NN}$:

(17) $$\tau_{NN}=G_{NN}\frac{1}{1+e^{-R}}$$

After the net output is applied to the process, a wait time is necessary for the process to respond to the control command $\tau_{NN}$. Then, the process’ error is computed considering the process’ output, and the weight coefficients of the neural network are adjusted. The backpropagation (BP) algorithm was applied in order to adjust the weight coefficients of the neural network, as explained below.

Backpropagation minimizes the quadratic function:

(18) $$E\left(t\right)=\frac{1}{2}\overset{t}{\underset{k=1}{\sum }}{\left({\tilde{\eta }}_{k}\right)}^{2}$$

The direction of the descending gradient is used to adjust the weight coefficients, so the gradient is only defined if *E(t)* is continuous and differentiable, thereby, the sigmoid function was used as the activation function

(19) $$-\nabla E\left(t\right)=-\left[\begin{array}{c} \frac{\partial E\left(t\right)}{\partial V_{j}}\\ \frac{\partial E\left(t\right)}{\partial W_{ji}} \end{array}\right]$$

Solving for the first term with the compound control signal error $e_{\tau_{\eta }}$ gives:

(20) $$\frac{\partial E\left(t\right)}{\partial V_{j}}=-\delta^{1}*H_{j}*\frac{\partial \tilde{\eta }}{\partial e_{\tau_{\eta }}}$$

where

(21) $$\delta^{1}=\tilde{\eta }*\frac{\tau_{NN}}{G_{NN}}\left(1-\frac{\tau_{NN}}{G_{NN}}\right)$$

Solving for the second term gives:

(22) $$\frac{\partial E\left(t\right)}{\partial W_{ji}}=-\delta_{j}^{2}*X_{i}*\frac{\partial \tilde{\eta }}{\partial e_{\tau_{\eta }}}$$

where

(23) $$\delta_{j}^{2}=\delta^{1}*V_{j}*H_{j}\left(1-H_{j}\right)$$

The term $\frac{\partial \tilde{\eta }}{\partial e_{\tau_{\eta }}}$ cannot be directly calculated, so it is replaced by:

(24) $$\frac{\partial \tilde{\eta }}{\partial e_{\tau_{\eta }}}=abs\left[\frac{\partial \tilde{\eta }}{\partial e_{\tau_{\eta }}}\right]*sign\left(\frac{\partial \tilde{\eta }}{\partial e_{\tau_{\eta }}}\right)$$

Instead of determining the $abs\left[\frac{\partial \tilde{\eta }}{\partial e_{\tau_{\eta }}}\right]$ term, it is incorporated as part of the learning coefficient “λ”, and the equations for adjusting the weight coefficients are:

(25) $$V_{j}\left(t+1\right)=V_{j}\left(t\right)+\lambda *sign\left(\frac{\partial \tilde{\eta }}{\partial e_{\tau_{\eta }}}\right)*\delta^{1}*H_{j}$$

(26) $$W_{ji}\left(t+1\right)=W_{ji}\left(t\right)+\lambda *sign\left(\frac{\partial \tilde{\eta }}{\partial e_{\tau_{\eta }}}\right)*\delta_{j}^{2}*X_{i}$$

Finally, the $sign\left(\frac{\partial \tilde{\eta }}{\partial e_{\tau_{\eta }}}\right)$ term is determined by experimental observations by introducing changes to the set-point of the controller for the closed-loop system performance.

## 4. Experimental Results

In this section, we present the experimental results of the proposed neuro-sliding controller (NSC) as well as those of the 2nd-SMC and the BP-NN controllers. The experimental setup is explained below.

### 4.1. Mini-ROV Architecture

**Figure 4:**
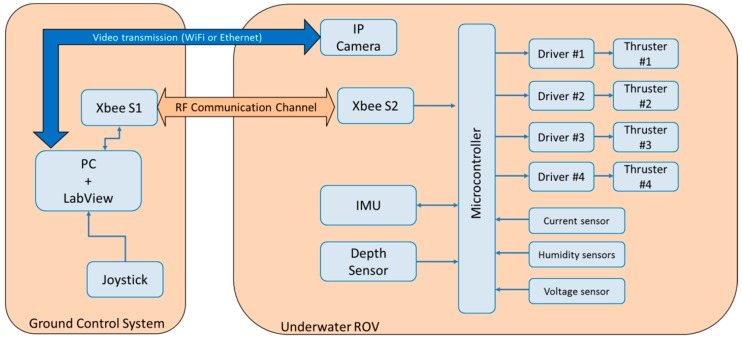
ROV electronic architecture.

Experiments were carried out in a salty water tank with a small-sized underwater ROV. A block diagram of the mini-ROV is shown in Figure 4. 

#### 4.1.1. Mechanical Architecture 

**Figure 5:**
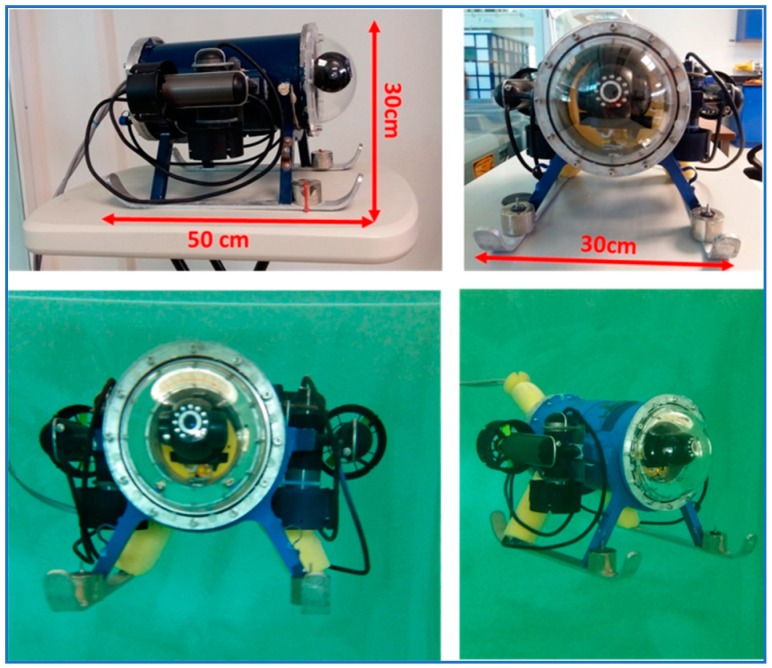
Small-sized sub-actuated underwater ROV used for the experiments.

The mini ROV’s body was principally made up of an aluminum 25 × 15 cm cylindrical pressure chamber wherein the electronic cards and the camera are placed. On both sides of the pressure chamber flanges with O’ ring slots mated in the bow with a polycarbonate transparent dome and in the stern with an aluminum cap with penetrators on it. The purpose of the penetrators is to allow the tether and the thruster wires to enter through the hull without water leaks. Beneath the pressure chamber, a frame with a pair of skis is screwed. This frame makes the vehicle’s center of gravity to below the center of buoyancy, thus, making the ROV intrinsically stable in both the pitch and roll angles. Figure 5 depicts some views of the mini ROV used for the experiments. 

**Figure 6:**
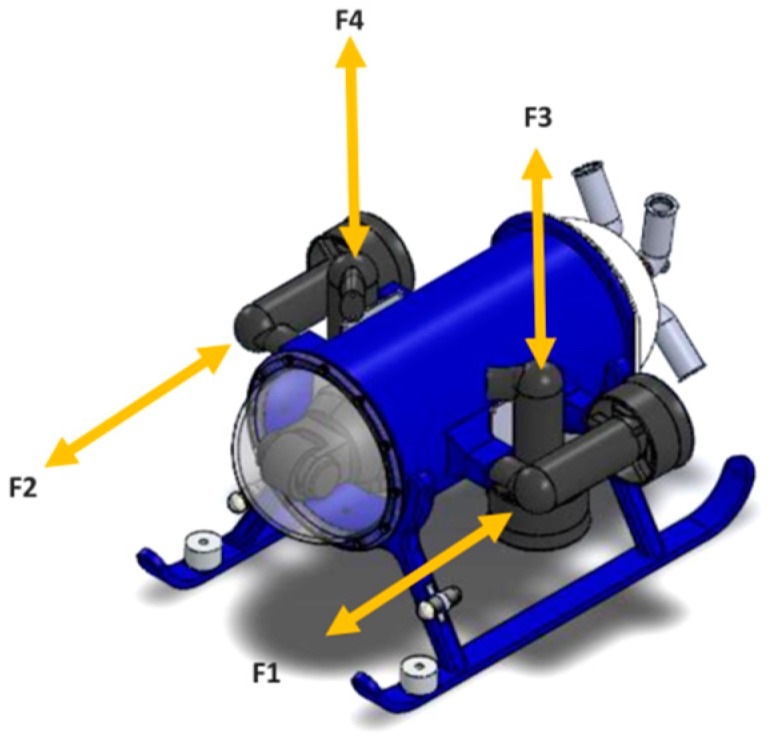
Location of the thrusters on the mini-ROV.

Four thrusters were attached to the vehicle, one horizontal and one vertical, on each side of the pressure chamber (port and starboard), as shown in Figure 6. Their main purpose is to allow the submarine to be actuated on its x and z axis.

#### 4.1.2. Electronic Architecture

The electronic architecture of the small sized ROV can be explained in two principal parts: the data acquisition system and the ROV-pilot interface.

The ROV’s cylindrical hull contained the data acquisition system where the central part was an AM3 × 8E ARM Cortex microcontroller embedded into an Arduino DUE board running at 84 MHz. Different kinds of sensors were connected to it that measured the actual status of the vehicle: A MS5837-30BA pressure sensor placed outside the hull with a penetrator that measured the hydrostatic pressure. As explained in [13], each milibar measured below the surface of water is equivalent to a centimeter of depth according to the formula that shows h as depth in meters [m], $h=\;\frac{P-P_{o}}{pg}$ where P is the hydrostatic pressure [Pa]; $P_{o}$ is the atmospheric pressure [Pa]; ρ is the water density $\left[\mathrm{kg}/m^{3}\right]$; and g is theacceleration of gravity $\left[m/s^{2}\right]$. This pressure sensor presents an accuracy of ±50 mbar in the operating pressure range of 0–6 bar, a sampling rate of 50 kHz, and is read by the I2C serial protocol.

Other sensors were connected to the microcontroller: a serial UART, PNI’s TCM-MB IMU (inertial measurement unit) that makes it possible to know the position of the ROV in the body-frame reference, two leak sensors to know if the electronics are in risk of damage because of water intrusion inside the hull, four hall-effect current sensors to read the status of each thruster during operation, a voltage sensor for brownout protection, an IP camera to stream live video to ground, and the thruster DC motor drivers (Pololu VNH5019).

The ROV pilot interface was a two screened PC with a LabView virtual instrument that displayed all of the sensors’ magnitudes received from the microcontroller. A joystick that worked as the manual controller of the vehicle was connected to the computer. One of the two screens connected to the PC displayed the video captured by the camera by a WiFi connection. The PC ran on a Windows operating system and was fitted with a Core i5 microprocessor (6300U@2.4 GHz) and 8 GB DDR3 RAM. The PC processed the input data coming from the ARM Cortex microcontroller and computed the summed control signal, which was in turn sent to the thruster drivers through the tether.

The communication between the submarine and the ROV pilot interface was through a pair of Xbee radio transmitter–receivers, one of which was on the dry end of the tether and another was connected to the PC that sent back and forward sentences with all the gathered data by the microcontroller. A WiFi modem was also connected to the tether in order to transmit live video to the PC.

### 4.2. Experiment Design

In this section, the description of the experiment for the three implemented controllers is given below. The complete experiment lasted 30 min in total (1800 s) and was divided into five tests, each test lasted six minutes and was composed of three stages: Stage 1-No perturbation, Stage 2-Perturbation, and Stage 3-No perturbation. The perturbation consists in manually adding and removing a weight of 1 kg (10% of the vehicle’s total weight) to and from the vehicle to evaluate the performance of the proposed controller. Each stage lasted two minutes (120 s); thus, a complete test lasted six minutes (360 s). In Stage 1, the vehicle was required to reach (follow) the set-point (trajectory) for a period of 120 s. In Stage 2, the weight was suddenly added to the vehicle for about 120 more s. In Stage 3, the weight was rapidly removed from the vehicle, which remained working for the next 120 s. In order to compare the performance of the controllers, the sequence was repeated five times to complete 1800 s in total.

**Remark** **1.** *The weight was chosen as long as it did not saturate the thrusters. The weight was gradually increased to the point where the thrusters were able to perform the task properly. It was found that a weight greater than 1 kg exceeded the thruster’s physical limits.*

Two sets of experiments were performed to control the vehicle’s depth. Only the depth was controlled, the remainder of the degrees of freedom was left uncontrolled. Thus, from Figure 2, η=z, $\eta_{d}=z_{d}$ and $\tilde{\eta }=z-z_{d}$.

(a) Set-point. The mini-ROV was required to reach a desired fixed depth, $z_{d}=0.3\;m$.
(b) Trajectory tracking. The vehicle was required to follow a sinusoidal trajectory:
  $$z_{d}=0.1\sin\left(t\right)+0.3\;\;m$$

**Figure 7:**
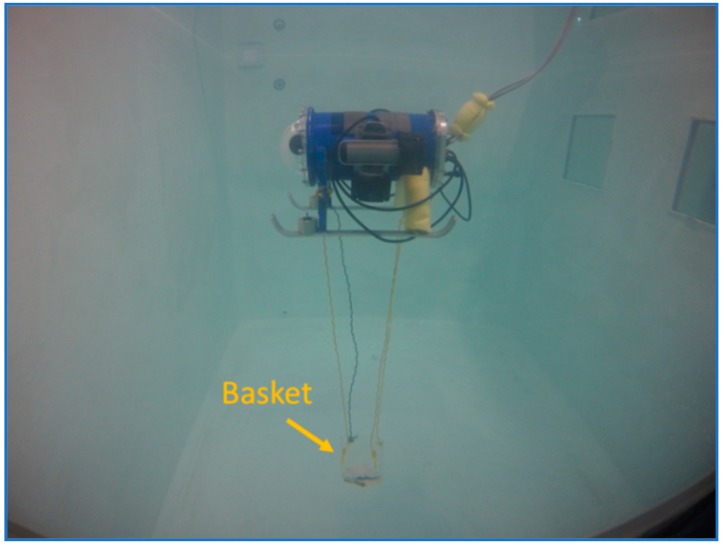
Basket attached to the mini-ROV used to hold the weight.

To add and remove the weight, a basket was attached to the bottom of the mini-ROV as shown in Figure 7.

Next, the experimental results are given.

### 4.3. Results

For comparison purposes, next, we present the experimental results of the three controllers addressed in this paper. The second order sliding mode control (2nd-SMC) was implemented with the feedback gains shown below in Table 1.

The backpropagation neural network control (BP-NN) was implemented with the learning coefficient set to 0.1. The neuro-sliding control (NSC) considered the feedback gains listed in Table 1 and a learning coefficient equal to 0.1.

To evaluate the performance of the proposed controllers, two criteria were used: (i) The square mean error (SME) that measures the position tracking error on average, and (ii) the effective voltage (RMS voltage) applied to the thrusters as a way to deduce how much energy is being consumed.

#### 4.3.1. Position Regulation ($z_{d}=0.3\;m$)

Table 2 shows the SME and the RMS values of the three controllers. Note that in the case of the NSC scheme, the total control signal was composed of two parts, a part given by the 2nd-SMC scheme and the other given by the BP-NN control. The sum of both signals gives the total voltage (100%) that can be supplied by the drivers to the thrusters (see Figure 2 for the major reference). 

**Figure 8:**
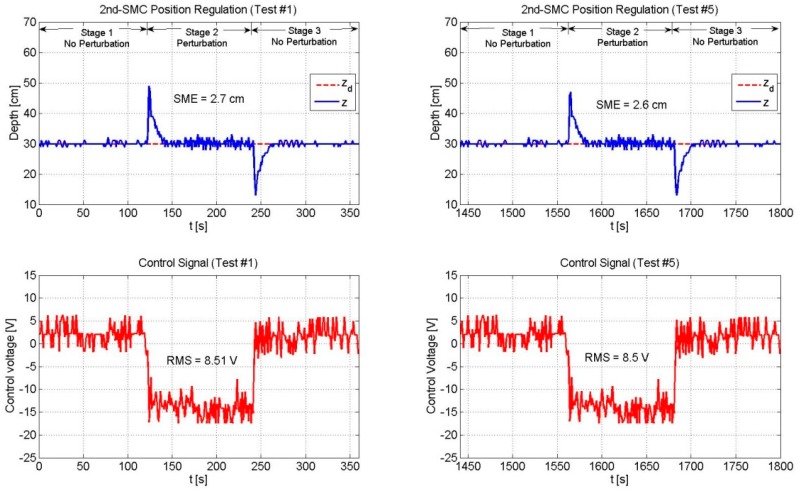
Performance of the 2nd-SMC scheme. The SME and the RMS values remained constant during the whole experiment.

**Figure 9:**
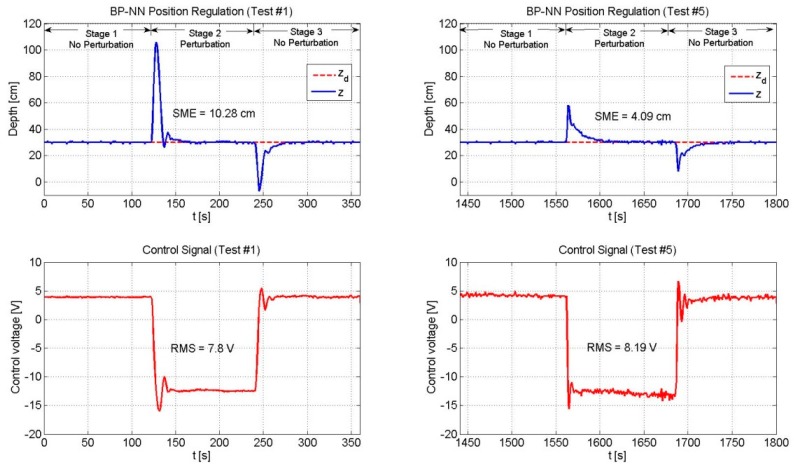
Performance of the BP-NN control. As time went by the neural network updated its weights online in order to drive the position error to zero. In the same fashion, the energy required to achieve a smaller SME increased.

**Figure 10:**
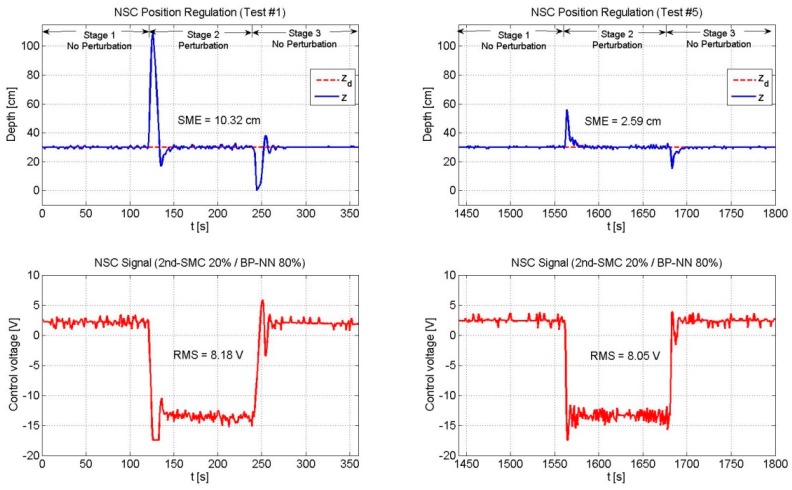
Performance of the NSC scheme (2nd-SMC 20%/BP-NN 80%). The BP-NN scheme appeared as the predominant control with a contribution of 80%. As the neural network adjusted its weights online, the SME decreased in time. The RMS voltage remained practically unchanged.

**Figure 11:**
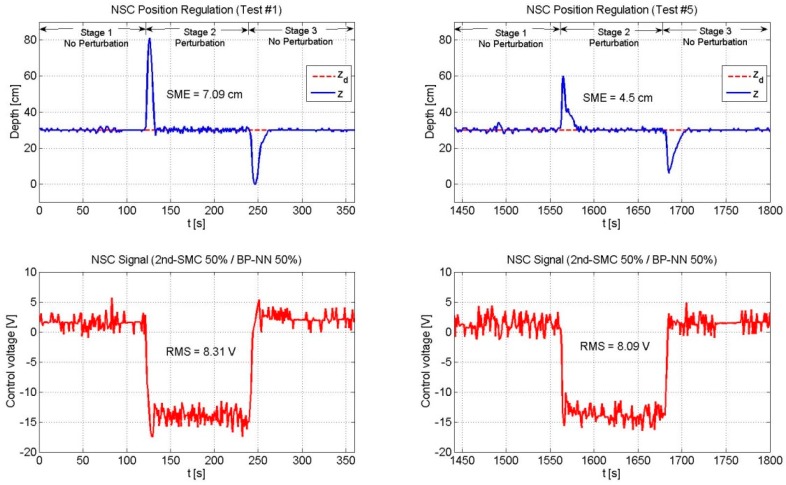
Performance of the NSC scheme (2nd-SMC 50%/BP-NN 50%). The overshoot showed a reduction and the BP-NN gradually reduced the SME value.

**Figure 12:**
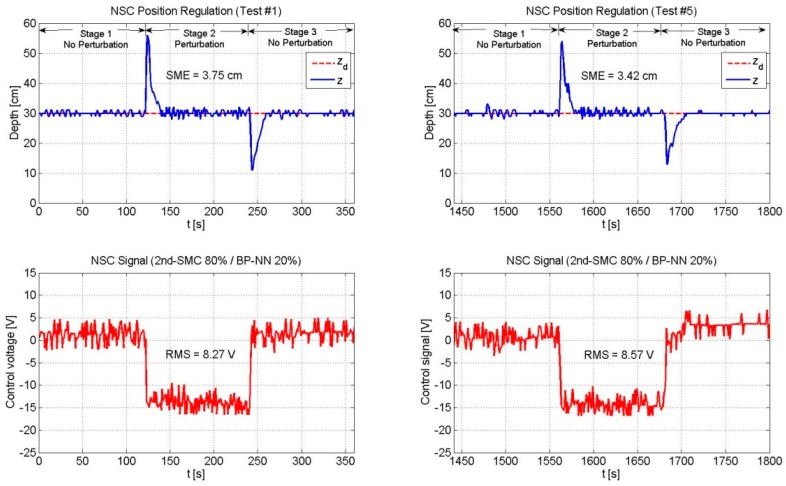
Performance of the NSC scheme (2nd-SMC 80%/BP-NN 20%). The 2nd-SMC scheme was the predominant control with a contribution of 80%. The SME exhibited a reduced value since the beginning; however, due to the low contribution of the BP-NN control, the SME value did not reflect a significant reduction by the end of the experiment. The RMS voltage remained practically unchanged throughout the experiment.

Figure 8, Figure 9, Figure 10, Figure 11 and Figure 12 depict the behavior of the system under the three controllers. The vehicle is required to control its depth position in $z_{d}=0.3\;m$. The whole experiment lasted 30 min. The plots located on the left side of the figure show the first six minutes of the experiment (Test #1). Similarly, the plots located on the right side depict the last six minutes of the experiment (Test #5).

According to Table 2 and Figure 8 and Figure 9, the 2nd-SMC scheme exhibited a better performance than the BP-NN scheme in terms of the SME value. Since the beginning of the experiment (Test #1) the 2nd-SMC showed a tight control of the depth variable and only at the moments when the load was added to and removed from the vehicle, the depth variable deviated for some seconds from the set-point. In both situations, the control law rapidly drove the position error to zero. This behavior remained constant throughout the experiment (until Test #5). Instead, the BP-NN presented a large overshoot in the first test when the load was added to and removed from the vehicle; however, as time went by, the neural network learnt the dynamics and compensated for the disturbance. The overshoots became smaller over time, and likewise, the SME value. With respect to the RMS voltage applied to the thrusters, the 2nd-SMC exhibited a more active control signal than the BP-NN scheme due to its high gain nature.

Throughout the experiment, the 2nd-SMC scheme presented a greater RMS value than the BP-NN control. However, it was noted that as long as the BP-NN’s SME decreased, its RMS voltage increased. In addition, it was observed that the 2nd-SMC scheme was capable of keeping the position error within certain limits throughout the experiment, but was not able to gradually reduce it as the BP-NN scheme did.

Next, Figure 10, Figure 11 and Figure 12 depict the NSC performance considering three cases: (1) 2nd-SMC 20%/BP-NN 80%; (2) 2nd-SMC 50%/BP-NN 50%; and (3) 2nd-SMC 80%/BP-NN 20%. According to Figure 2, each control signal was multiplied by its respective scaling factor $G_{NN}$ and $G_{SMC}$ to generate the BP-NN control signal ($\tau_{NN})$ and 2nd-SMC signal $\left(\tau_{SMC}\right)$, respectively. The scaled control signals were summed to generate the 100% control voltage that the driver could supply to the thrusters. 

Figure 10 depicts the experimental results of the NSC scheme (2nd-SMC 20%/BP-NN 80%). Since the control voltage is mainly composed of the BP-NN part, the SME value was very similar to that of the pure BP-NN as shown in Figure 9 for Test #1. However, after some time, in Test #5, the SME represented 63.3% of the pure BP-NN’s SME value, which means that the neural network is working properly.

Figure 11 depicts the experimental results of the NSC scheme (2nd-SMC 50%/BP-NN 50%). In this case, the vehicle exhibited a smaller overshoot when compared to that of the previous case, mainly because of the 2nd-SMC action. The SME gradually decreased, thanks to the BP-NN action. Note that inTest #5, the SME value represented 64.3% of the SME value reported in Test #1; which means that the BP-NN acts as a fine control that correctly adjusts its weight coefficients to overcome the perturbation’s effects. 

Figure 12 depicts the experimental results of the NSC scheme (2nd-SMC 80%/BP-NN 20%). In this case, the 2nd-SMC had a major contribution to the total control voltage. Compared to the previous cases, the SME values presented a significant reduction. Nevertheless, due to the low contribution of the BP-NN control, the SME value in Test #5 showed no significant reduction with respect to that of Test #1; instead, the RMS value experienced an increase in its value due to the 2nd-SMC action. 

Next, the experimental results of the NSC scheme following a trajectory are given.

#### 4.3.2. Tracking Trajectory ($z_{d}=0.1\sin\left(t\right)+0.3\;m$)

**Figure 13:**
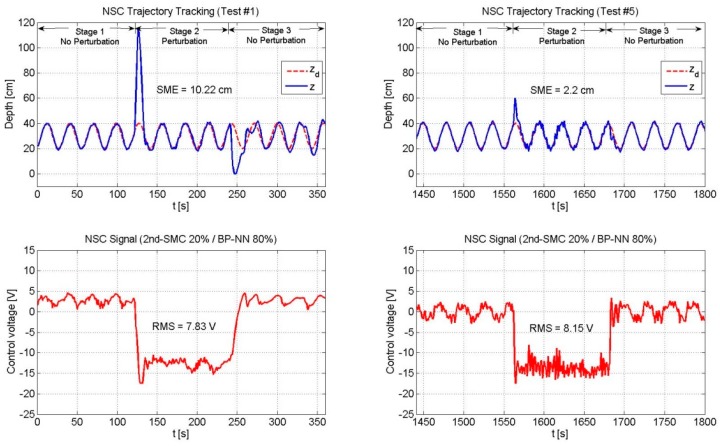
Performance of the NSC scheme (2nd-SMC 20%/BP-NN 80%).

**Figure 14:**
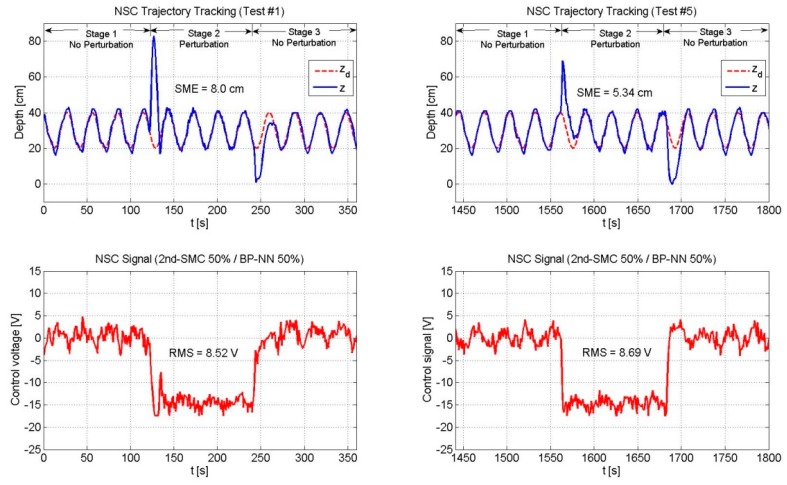
Performance of the NSC scheme (2nd-SMC 50%/BP-NN 50%).

**Figure 15:**
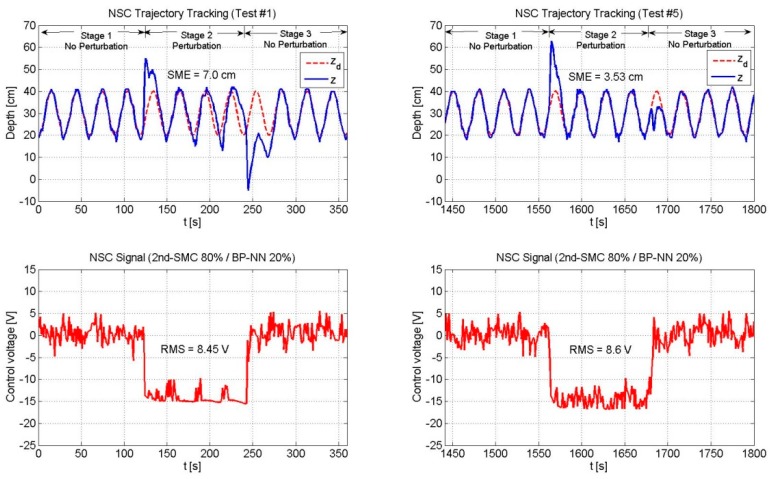
Performance of the NSC scheme (2nd-SMC 80%/BP-NN 20%).

Table 3 shows the SME and the RMS values of the three controllers. Figure 13, Figure 14 and Figure 15 depict only the experimental results of the NSC scheme (for the sake of space, figures related to the 2nd-SMC and BP-NN schemes are omitted).

In this subsection, the experimental results of the NSC scheme are given. The mini-ROV was required to follow a sinusoidal trajectory under the three NSC combinations described above. 

Figure 13, Figure 14 and Figure 15 depicts the performance of the NSC scheme under the three combinations. Two general aspects to highlight are: (1) The overshoot was large at the beginning and decreased over time by virtue of the BP-NN control action, and (2) As long as the SME decreased, the RMS value increased.

## 5. Conclusions

According to the experimental results, it was observed that the 2nd-SMC scheme drove the position error to zero in a robust manner while keeping a small overshoot with respect to the BP-NN response. The BP-NN control had the ability to estimate the vehicle’s hydrodynamic variations and to compensate for perturbations by adjusting its weights online in order to gradually drive the position error to zero. However, the weight adjustment was relatively slow because it took several iterations before the weight values converged to a steady state, which can be a disadvantage if the perturbation changes rapidly over time. If the perturbation changes faster than the weight adjustment, the BP-NN control may not be able to compensate for it completely. Thus, a smooth but robust controller like a 2nd-SMC is required. Hence, the combination of both controllers results in a more robust and versatile controller. According to the SME and the RMS values, it can be noted that the best performance was accomplished when the NSC scheme was mainly composed of either the 2nd-SMC or the BP-NN action. The 50/50 percent case exhibited good performance; however, we observed that in the whole cases, the SME value in Test #5 was greater than in the other cases. In light of the latter, we suggest choosing cases where one of the controllers has a major contribution, that is, $G_{SMC}>G_{NN}$ or $G_{NN}>G_{SMC}$, depending on the task, the desired performance, and the operating conditions.

Future work is to find a method to determine (offline or online) the set of scaling factors $G_{SMC}$, $G_{\mathrm{NN}}$ that achieve the best performance of the system. Experiments considering controlled currents and the heading control remain an opportunity for future research.

## Figures and Tables

**Table 1:** 2nd-SMC feedback gains.

Gain	Value	Gain	Value
α	15	$K_{i}$	43
κ	43	$K_{d}$	0.7

**Table 2:** Square mean error (SME) and effective value (RMS) comparison for a set-point of $z_{d}=0.3\;m$.

Control Law	Control Signal (%)		Test #1		Test #5	
	2nd-SMC	NN	SME [cm]	RMS [volts]	SME [cm]	RMS [volts]
2nd-SMC	100	-	2.7	8.51	2.6	8.5
BP-NN	-	100	10.28	7.8	4.09	8.19
NSC	20	80	10.32	8.18	2.59	8.05
NSC	50	50	7.09	8.31	4.5	8.09
NSC	80	20	3.75	8.27	3.42	8.57

**Table 3:** Square mean error (SME) and effective value (RMS) comparison for a trajectory of $z_{d}=0.1\sin\left(t\right)+0.3\;m$.

Control Law	Control Signal (%)		Test #1		Test #5	
	SMC	NN	SME [cm]	RMS [volts]	SME [cm]	RMS [volts]
2nd-SMC	100	-	2.51	8.36	2.51	8.36
BP-NN	-	100	10.78	7.56	1.82	8.0
NSC	20	80	10.22	7.83	2.2	8.15
NSC	50	50	8.0	8.52	5.34	8.69
NSC	80	20	7.0	8.45	3.53	8.6
